# Aerospace Mutagenized Tea Tree Increases Rhizospheric Microorganisms, Enhances Nutrient Conversion Capacity and Promotes Growth

**DOI:** 10.3390/plants14070981

**Published:** 2025-03-21

**Authors:** Weiting Cheng, Yulin Wang, Yuhua Wang, Lei Hong, Miaoen Qiu, Yangxin Luo, Qi Zhang, Tingting Wang, Xiaoli Jia, Haibin Wang, Jianghua Ye

**Affiliations:** 1 College of Horticulture, Fujian Agriculture and Forestry University, Fuzhou 350002, China; 2 College of Tea and Food, Wuyi University, Wuyishan 354300, China; 3 College of Life Science, Longyan University, Longyan 364012, China

**Keywords:** tea tree, aerospace mutagenesis, characteristic microorganisms, nutrient conversion, growth

## Abstract

The utilization of aerospace mutagenesis in plant breeding is a novel, efficient technology. This study investigates the effects of aerospace mutagenesis on tea tree growth, soil nutrient conversion, and soil microbial community structure and function. The results showed that aerospace mutagenized tea trees showed increased leaf area, 100-bud weight, and yield. The rhizosphere soil of mutagenized tea tree displayed an increase in microorganisms, enhanced carbon and nitrogen cycling capacity, and significant increases in nutrient conversion and antioxidant enzyme activities. In addition, the content of available nutrients was also increased. Aerospace mutagenesis showed an increase in the abundance of soil-characteristic microorganisms (*Solirubrobacterales bacterium*, *Capillimicrobium parvum*, *Mycobacterium colombiense*, *Mycobacterium rhizamassiliense*, and *Conexibacter woesei*), and enhancement of the intensity of metabolic pathways, glyoxylate and dicarboxylate metabolism, biosynthesis of secondary metabolites, microbial metabolism in diverse environments, carbon metabolism, fatty acid metabolism, carbon metabolism, biosynthesis of amino acids, and biosynthesis of cofactors of soil microorganisms. Interaction network and partial least squares structural equation modeling (PLS-SEM) equation analysis showed that after aerospace mutagenesis, soil-characteristic microorganisms positively affected soil microbial functions, soil microbial biomass carbon and nitrogen, respiration intensity, and soil enzyme activities; furthermore, it improved available nutrient content and tea tree growth. This study provides an important reference for the cultivation and management of aerospace mutagenized tea trees and microbial regulation of tea tree growth.

## 1. Introduction

Plant breeding facilitates the development of new plant varieties with superior characteristics and is an important research area in agricultural production [1]. Traditional breeding methods, e.g., crossbreeding selection, pure line selection, etc., are effective in selecting a large number of plants with superior traits, but selection takes an extended period [2]. Aerospace mutagenesis is a novel technology for efficient plant breeding. Space has a more specialized environment, for example, higher radiation, microgravity, extreme temperatures, vacuum, etc. [3]. Plant seeds are highly susceptible to changes in their gene regulation and genetic behavior under the combined influence of special spatial environmental factors, which in turn can alter the genetic and physiological characteristics of the plant and affect subsequent planting and growth [4]. Therefore, aerospace mutagenesis has been utilized for rapid screening of plant germplasm resources with superior traits [5]. For example, the Chinese Academy of Sciences developed two new tomato varieties with high yield and disease resistance using aerospace mutagenesis [6]. The Tasly group used aerospace mutagenesis to effectively increase the content of medicinal components of *Salvia miltiorrhiza* and bred high-quality *Salvia miltiorrhiza* [7]. It is evident that aerospace mutagenesis plays a crucial role in the selection and breeding of new plant varieties.

Wuyishan in Fujian Province is one of the most important tea-producing areas in China and the birthplace of black and oolong tea. Dahongpao (*Camellia sinensis*) is one of the major cultivated tea tree varieties in Wuyishan [8]. China started late in tea tree aerospace breeding, from 2003 until now. China has conducted 12 batches of aerospace mutagenesis breeding of tea trees, and Dahongpao is the only batch of Wuyi Rock Tea for aerial mutagenesis. A previous study found that aerospace mutagenesis of Dahongpao seedlings resulted in significant changes in branching and leaf morphology [9]. The aerospace mutagenesis of Dahongpao resulted in more regular serrations and tighter leaf edge arrangement, smoother leaf surface, and a significant increase in leaf area and hundred bud weight, and compared with control, mutagenized tea trees had high chlorophyll content, had a better photosynthetic capacity and resistance, and higher yield [10]. It can be seen that Dahongpao, after aerospace mutagenesis, has undergone obvious changes in growth and physiology, and its excellent traits have been emphasized. The in-depth revelation of the mechanism of changes in tea tree growth after aerospace mutagenesis is of great importance for planting and promoting new varieties of high-quality Dahongpao after mutagenesis.

Plants cannot grow without the supply of nutrients, and soil is a storehouse of nutrients [11]. Plants absorb nutrients through their roots and then transfer them to each tissue that supports their growth [12]. The level of soil nutrients, especially available nutrients, directly affects nutrient uptake and plant growth [13]. Microorganisms play a crucial role in soil nutrient conversion, increasing soil available nutrient content, enhancing nutrient uptake by plants, and promoting plant growth [14]. Secondly, microorganisms are not only involved in soil nutrient cycling and organic matter conversion, but can also modify soil habitat, positively affect plant root growth, positively regulate nutrient uptake capacity of plants, and promote plant growth [15,16]. For example, *Achromobacter*, *Azotobacter*, *Bacillus*, *Rhizobium*, etc., play crucial roles in soil nutrient cycling, soil fertility preservation, and organic matter decomposition and can be used as plant growth promoters [17]. *Frankia* accelerates the mineralization of phosphorus and potassium in rhizosphere soil after plant root colonization, enhances soil nutrient effectiveness, and is a reciprocal symbiosis with plants [18]. It is clear that the variety of microorganisms present in plant rhizosphere soil, together with their respective functions, is closely related to soil nutrient conversion and plant development [19]. It was hypothesized that, after aerospace mutagenesis, the rhizosphere soil microbial diversity and its function in tea trees were significantly altered, which in turn affected the soil’s nutrient conversion capacity and the tea tree’s uptake of nutrients and affected tea tree growth. In-depth excavation of the characteristic microorganisms and their effects on tea tree growth in rhizosphere soil after aerospace mutagenesis is of great importance for the planting management of new varieties of aerospace mutagenized tea trees and the use of microorganisms to regulate the growth of tea trees. Therefore, the purpose of this study was to investigate changes in the diversity of rhizosphere soil microorganisms of aerospace mutagenized tea trees and their effects on soil nutrient cycling and tea tree growth.

## 2. Results and Discussion

### 2.1. Effect of Aerospace Mutagenesis on the Growth of Tea Trees

**Figure 1:**
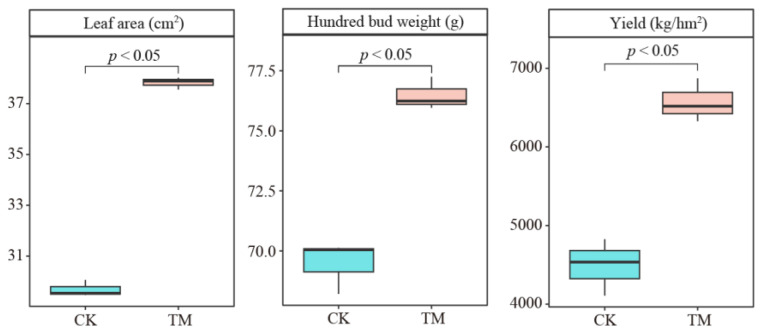
Effect of aerospace mutagenesis on the growth of tea trees. Note: CK is tea trees without aerospace mutagenesis; TM is tea trees with aerospace mutagenesis.

This study found that aerospace mutagenesis had a significant impact on tea tree growth (Figure 1). Specifically, tea trees that underwent no aerospace mutagenesis (CK) exhibited leaf area, hundred bud weight, and yield of 29.69 cm^2^, 69.46 g, and 4490.67 kg/hm^2^, respectively. In contrast, tea trees subjected to aerospace mutagenesis (TM) showed leaf area, hundred bud weight, and yield of 37.82 cm^2^, 76.48 g, and 6573.01 kg/hm^2^, respectively. Following aerospace mutagenesis, alterations in plant genetic and gene expression can easily affect their physiological characteristics and plant growth [20]. These findings suggest that aerospace mutagenesis effectively enhances leaf area, hundred bud weight, and tea yield of tea trees.

### 2.2. Effects of Aerospace Mutagenesis on Nutrient Contents, Enzyme Activities, and Microbial Physiological Indices of the Rhizosphere Soil of Tea Trees

**Figure 2:**
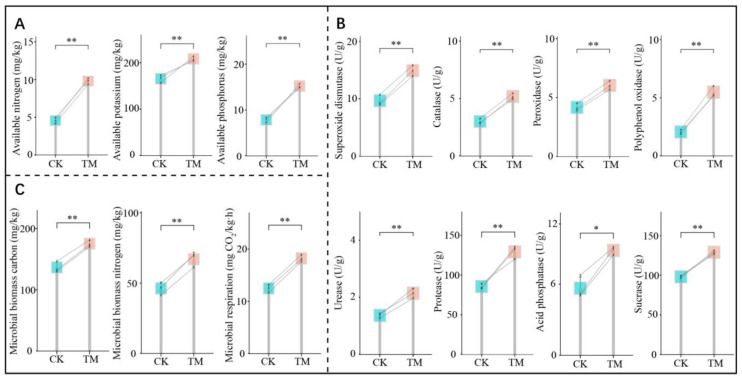
Effect of aerospace mutagenesis on rhizosphere soil nutrient content, enzyme activity, and microbial physiological indices of tea trees. Note: CK is tea trees without aerospace mutagenesis; TM is tea trees with aerospace mutagenesis; (**A**) Soil available nutrient contents; (**B**) Soil nutrient cycling and resistance-related enzyme activities; (**C**) Soil microbial biomass carbon and nitrogen and respiration intensity; * and ** indicate that the differences between the two samples reached the *p* < 0.05 and *p* < 0.01 levels, respectively.

Soil is rich in nutrients, but many of these nutrients are in organic form and must be converted into inorganic form to be absorbed and used by plants. Therefore, the content of available nutrients in the soil has a direct impact on plant growth [21]. In this study, it was found that, after aerospace mutagenesis, the rhizosphere soil of tea trees subjected to mutagenesis (TM) had significantly higher available nutrient content than the control (CK). Specifically, the available nitrogen, available phosphorus, and available potassium contents in TM were 9.79, 15.24, and 208.12 mg/kg, respectively, while those in CK were 4.56, 7.85, and 165.40 mg/kg, respectively (Figure 2A). Analysis of soil enzyme activities further revealed that aerospace mutagenesis significantly enhanced the activities of enzymes related to nutrient cycling and antioxidants in tea tree rhizosphere soil (Figure 2B). Activities of urease, protease, acid phosphatase, and sucrase associated with nutrient cycling in TM rhizosphere soils were 2.14, 129.19, 8.83, and 128.88 U/g, respectively, compared to 1.37, 85.60, 5.64, and 98.01 U/g in CK soils. Additionally, the activities of soil superoxide dismutase, peroxidase, catalase, and polyphenol oxidase, which are related to antioxidants, were 14.93, 6.02, 5.18, and 5.49 U/g for TM and 9.67, 4.17, 3.01, and 2.06 U/g for CK, respectively.

In addition, this study also measured microbial physiological indices of tea tree rhizosphere soils. The results showed that soil microbial biomass carbon, microbial biomass nitrogen, and microbial respiration in TM rhizosphere soils were 175.14 mg/kg, 66.95 mg/kg, and 18.24 mg CO_2_/kg·h, respectively, compared to 136.52 mg/kg, 46.21 mg/kg, and 12.47 mg CO_2_/kg·h in CK soils (Figure 2C). These values were significantly greater in TM than in CK. The activity of soil enzymes related to nutrient cycling is crucial for assessing soil fertility [22], and higher activity promotes soil organic matter degradation and enhances the biotransformation of soil nutrients such as nitrogen, phosphorus, and other elements, thereby increasing the soil’s available nutrient content [23,24]. On the other hand, soil antioxidant enzymes contribute to improving soil texture, maintaining soil health, and providing an enabling environment for plant root growth and microbial colonization [25]. Therefore, it is evident that after aerospace mutagenesis, the rhizosphere soil of the tea tree exhibited an increase in microbial numbers, enhanced carbon and nitrogen cycling capacity, significantly elevated activity of nutrient conversion and antioxidant-related enzymes, and increased soil available nutrient content, all of which were more favorable to the growth of the tea tree.

### 2.3. Soil Microbial Macro-Genome Sequencing Analysis

The rhizosphere soil macro-genomic analysis of both CK and TM yielded a total of 38.06 G of clean data following sequencing with the Illumina platform (Table S1). The assembled clean data resulted in 1,425,145 contigs with a cumulative length of 1,123,414,004 bp (Table S2). ORF prediction of these contigs identified a total of 462,299 genes, spanning a total length of 636.34 Mbp and averaging 437.51 bp in length (Table S3). Following macro-genome analysis, it is essential to assess the stability of the genes, typically through the analysis of core and pan gene dilution curves [26]. As the sample volume increases, the closer the gene dilution curves of both core and pan genes approach smoothness, the more stable the number of detected genes becomes, with minimal newly added genes. This means that sequencing results have largely captured genetic information within the soil [27]. In this study, the number of core genes gradually decreased and plateaued when the number of samples reached 5, and the number of pan genes gradually increased and stabilized at this point (Figure S1). These findings suggest that macro-genome sequencing results of CK and TM accurately represent microbial communities within rhizosphere soil and can be used for further comprehensive analysis.

### 2.4. Effect of Aerospace Mutagenesis on the Microbial Diversity of Rhizosphere Soil of Tea Trees

**Figure 3:**
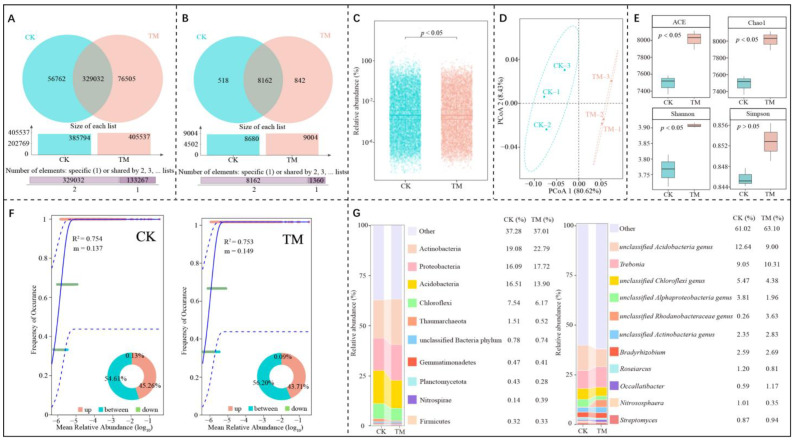
Rhizosphere soil microbial diversity analysis of Dahongpao tea tree. Note: CK is tea trees without aerospace mutagenesis; TM is tea trees with aerospace mutagenesis; (**A**) Vane diagram analysis of soil genes; (**B**) Vane diagram analysis of soil microorganisms; (**C**) Analysis of differences in microbial abundance; (**D**) PCoA analysis of β-diversity indices; (**E**) Analysis of α-diversity indices, including ACE, Chao1, Shannon, and Simpson indicators; (**F**) Analysis of microbial neutral community model; (**G**) Microorganisms in the top ten abundance proportions in the rhizosphere soils of the CK and TM at the phylum and genus level.

Macro-genome sequencing of rhizosphere soils from both CK and TM revealed the presence of a total of 462,299 genes, of which 329,032 genes were shared by CK and TM, 56,762 genes were specific to CK, and 76,505 genes were specific to TM (Figure 3A). Species annotation with the obtained genes resulted in a total of 9522 microbial species, of which 8162 were common to CK and TM, 518 were unique to CK, and 842 were unique to TM (Figure 3B). Further analysis of the specy abundance of microorganisms revealed that CK was significantly different from TM (*p* < 0.05) (Figure 3C). These findings suggest that aerospace mutagenesis induced significant alterations in rhizosphere soil microorganisms of tea trees, ultimately affecting the microbial community structure.

Alterations in the microbial community structure, including shifts in microbial diversity and abundance, have implications for the stability of the microbial community and the extent of its dispersion [28]. To evaluate the diversity and abundance of microorganisms within a sample, α-diversity indices (includes ACE, Chao1, Shannon, and Simpson) are employed, whereas β-diversity indices are utilized to compare the diversity and abundance of microorganisms across different samples [29,30]. In this study, the results indicated a statistically significant difference in the β-diversity indices between CK and TM, with the two principal components effectively distinguishing between the two, contributing a total of 89.05% to the differentiation (Figure 3D). Additionally, the α-diversity indices analysis revealed that the ACE, Chao1, Shannon, and Simpson indices for TM were higher compared to CK, with the ACE, Chao1, and Shannon indices for TM being significantly greater than those of CK (*p* < 0.05) (Figure 3E). These findings suggest that there was a significant change in microbial community diversity between CK and TM, with TM exhibiting significantly greater species diversity and richness than CK.

Furthermore, this study employed the neutral community model to delve into the microbial community structure. The results indicated that the R^2^ values for CK and TM were 0.754 and 0.753, respectively, suggesting they closely resembled the neutral model (Figure 3F). The migration rates of TM and CK were 0.149 and 0.137, respectively. The microbial neutral community model is a mathematical model based on neutral theory to describe and predict patterns of species diversity and community structure in microbial communities, and its mobility is an important indicator to assess the complexity and dispersion of microbial community structure. The greater the mobility, the lower the restriction of microbial dispersal, and the greater the dispersion of the community, and the more complex it is [31]. It can be seen that the microbial community structure of TM is more decentralized and complex than CK. A deeper microbial community abundance in CK and TM revealed that at the gate level, Actinobacteria (19.08%), followed by Acidobacteria (16.51%), followed by Proteobacteria (16.09%) accounted for the largest proportion in CK, while in TM the largest proportion was accounted for by Actinobacteria (22.79%), followed by Proteobacteria (17.72%), followed by Acidobacteria (13.90%) (Figure 3G). At the genus level, the three most abundant genera in both CK and TM were *Acidobacteria genus*, *Trebonia*, and *Chloroflexi genus*, with abundances of 12.64%, 9.05%, 5.47% in CK, and 9.00%, 10.31%, 4.38% in TM, respectively. These findings underscore that aerospace mutagenesis significantly altered the rhizosphere soil microbial community structure of tea trees, enhancing microbial diversity and richness, increasing the diffusion ability of the microbial community, and making its community structure more complex. However, the dominant microbial populations remained relatively similar, with only some differences in abundance.

### 2.5. Modular Network Node Analysis of Microorganisms in the Rhizosphere Soil of Tea Tree

**Figure 4:**
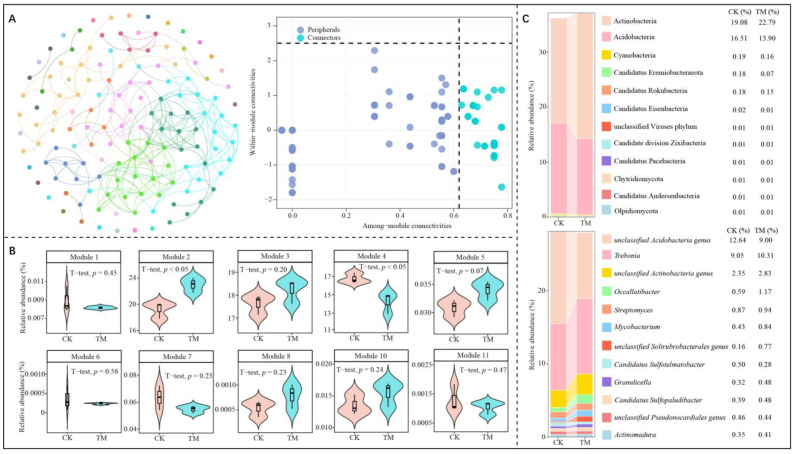
Modular network node analysis of rhizosphere soil microorganisms of Dahongpao tea tree. Note: CK is tea trees without aerospace mutagenesis; TM is tea trees with aerospace mutagenesis; (**A**) Modular interaction network and keystone node analysis of microorganisms; (**B**) Keystone node microorganism abundance analysis of different modules; (**C**) Top ten microorganisms in terms of abundance after grouping from phylum and genus level for module 2 and module 4.

**Figure 5:**
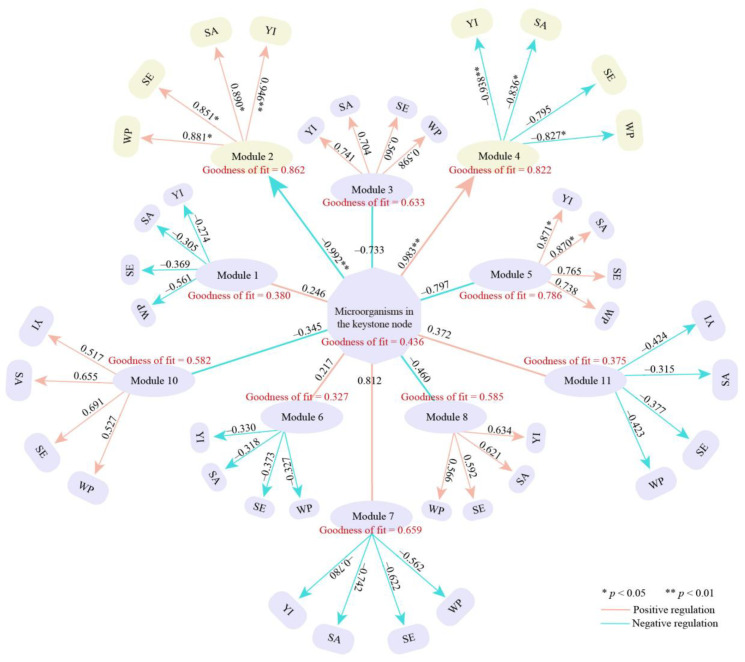
PLS-SEM equations of different modules of microorganisms with soil nutrient content, soil enzyme activity, microbial physiological indices, and tea tree growth indices. Note: YI is tea tree growth indices; SA is soil available nutrient content; SE is soil enzyme activity; WP is soil microbial physiological indices.

Different types of microorganisms in a microbial community interact with each other under different environmental conditions, leading to coexistence and symbiosis [32]. Therefore, analyzing the interactions between different microbial groups is important for clarifying the functional roles of microorganisms in the environment [33]. Microbial network analysis is an important method for identifying microorganisms with important roles in flora and their symbiotic relationships with other microorganisms [34]. A modular interaction network is a commonly used technique in microbial network analysis, which classifies microbial taxa modularly and obtains keystone nodes with high connectivity between different modules, thus obtaining microorganisms with direct relationships [35]. Therefore, this study further categorized these microorganisms and analyzed them using a modular network and found that the above-identified microorganisms could be categorized into 139 phyla, which, after modularization, could be classified into 10 modules. A total of 38 keystone nodes with high connectivity were obtained from the analysis of the topological roles of the nodes of these 10 modular networks (Connector, Pi > 0.62, Zi < 2.50) (Figure 4A). Further analysis of the microbial abundance of keystone nodes in the 10 modules revealed that only the microbial abundance of module 2 and module 4, out of the 10 modules, differed significantly between CK and TM, while the rest of the 8 modules had no significant differences between the two (Figure 4B). The abundance of microorganisms in module 2 of TM was significantly greater than that of CK, whereas the abundance of microbial in module 4 of TM was significantly less than that of CK. Accordingly, this study combined the microorganisms in module 2 and module 4 to analyze microbial communities with important roles in CK and TM, and the results indicated that the two modules corresponded to 3665 microorganisms. The three most abundant microorganisms in CK and TM at the phylum level were Actinobacteria, Acidobacteria, and Cyanobacteria, with abundances of 19.08%, 16.51%, and 0.19% in CK and 22.79%, 13.90%, and 0.16% in TM, respectively (Figure 4C). At the genus level, the three most abundant genera in CK and TM were *Acidobacteria genus*, *Trebonia*, and *Actinobacteria genus*, with abundances of 12.64%, 9.05%, 2.35% in CK, and 9.00%, 10.31%, 2.83% in TM, respectively. These findings suggest that aerospace mutagenesis had a significant impact on the microbial community structure in the rhizosphere soil of tea trees, particularly on the keystone node microorganisms in modules 2 and 4 that played crucial roles, which in turn may affect soil micro-ecosystem and tea tree growth. Accordingly, this study further constructed PLS-SEM equations of 38 keystone node microorganisms with high connectivity in 10 modules with different module microorganisms, tea tree growth indices, soil nutrient content, enzyme activity, and microbial physiological indices. The results revealed that these keystone node microorganisms could significantly regulate module 2 and module 4 and did not reach a significant level for the remaining modules. Secondly, module 2 could significantly regulate tea tree growth indices, soil nutrient content, enzyme activity, and microbial physiological indices, while module 4 could significantly regulate tea tree growth indices, soil nutrient content, and microbial physiological indices (Figure 5). These findings suggested that after aerospace mutagenesis, the keystone node microorganisms in module 2 and module 4, which were the main microorganisms distinguishing CK and TM, were the key microorganisms affecting available nutrient contents, soil enzyme activities, microbial physiological indices, and tea tree growth.

### 2.6. Screening of Characteristic Microorganisms in the Rhizosphere Soil of Tea Trees After Aerospace Mutagenesis

**Figure 6:**
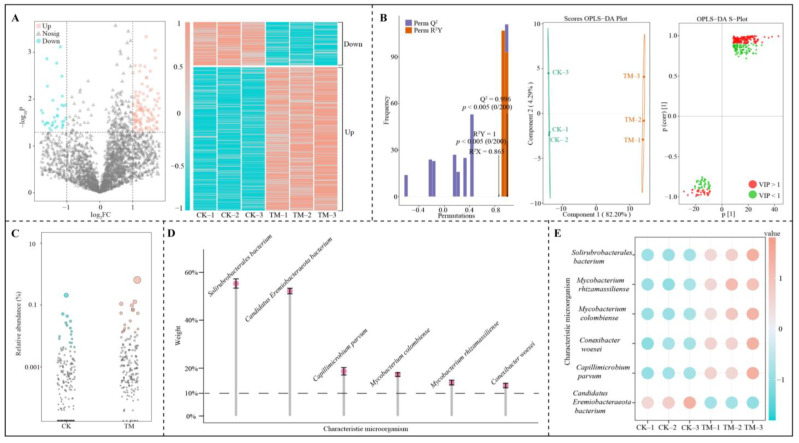
Screening of characteristic microorganisms in the rhizosphere soil of Dahongpao tea trees. Note: CK is tea trees without aerospace mutagenesis; TM is tea trees with aerospace mutagenesis; (**A**) Volcano plot screening and abundance analysis of differential microorganisms between CK and TM; (**B**) OPLS-DA model construction of CK and TM to screen key differential microorganisms; (**C**) Bubble feature diagram screening of characteristic microorganisms distinguishing between CK and TM; (**D**) TOPSIS analysis of the contribution of characteristic microorganisms in distinguishing between CK and TM; (**E**) Abundance analysis of characteristic microorganisms.

This study was conducted with 15 keystone node microorganisms with high connectivity in module 2 and module 4 to screen for characteristic microorganisms that were significantly different between CK and TM. A total of 3665 microorganisms were associated with the 15 keystone nodes. An analysis using volcano plots revealed that 289 microorganisms exhibited significant differences between CK and TM (Figure 6A). Specifically, the abundance of 222 microorganisms was significantly higher in TM compared to CK, while the abundance of 67 microorganisms was significantly lower in TM compared to CK. An OPLS-DA model was constructed using 289 microorganisms with significant differences, enabling the screening of key microorganisms distinguished in CK and TM. Firstly, the study evaluated the fit (R^2^Y) and predictability (Q^2^) of the OPLS-DA model. The results indicated that both the fit and predictability of the model were statistically significant (*p* < 0.005). Secondly, the model successfully distinguished between CK and TM, with an inter-group difference of 82.20% and an intra-group difference of less than 4.29%. Subsequently, the variable importance projection values (VIP) of various microorganisms in differentiating CK from TM were derived from the model’s S-plot. A total of 168 key microorganisms with VIP exceeding 1 were identified (Figure 6B). To further screen the characteristic microorganisms that distinguished CK from TM, a bubble feature map was employed, resulting in the identification of 20 significantly different characteristic microorganisms (Figure 6C). Based on the above analysis, this study deeply analyzed the contribution of the characteristic microorganisms that distinguished CK from TM by TOPSIS. The results found that only six microorganisms contributed more than 10% to distinguishing CK from TM, namely *Solirubrobacterales bacterium*, *Candidatus Eremiobacteraeota bacterium*, *Capillimicrobium parvum*, *Mycobacterium colombiense*, *Mycobacterium rhizamassiliense* and *Conexibacter woesei* (Figure 6D). The abundance analysis of the above six characteristic microorganisms showed that only the abundance of *Candidatus Eremiobacteraeota bacterium* was significantly smaller in TM than in CK, whereas the remaining five microorganisms were all significantly larger in TM than in CK (Figure 6E). Aerospace mutagenesis alters plant physiology and metabolism as well as the type and amount of plant root secretions [36]. Plant root secretions have a certain induction or avoidance effect on microorganisms, which in turn changes the microbial diversity of the rhizosphere soil and affects soil microbial function and nutrient cycling [37]. It can be seen that after aerospace mutagenesis, the changes in microbial diversity and abundance in TM rhizosphere soil may be related to its root secretion, and this change may lead to the influence of soil nutrient cycling.

*Solirubrobacterales bacterium* has been reported to be significantly associated with soil carbon metabolism, favoring an increase in soil carbon metabolism [38]. *Capillimicrobium parvum* improves nitrogen conversion and enhances the content of soil available nitrogen [39]. *Mycobacterium colombiense* and *Mycobacterium rhizamassiliense* are bacteria that use organic matter as carbon sources and energy and can break down organic matter into usable available nutrients, especially carbon and phosphorus [40,41]. *Conexibacter woesei* enhances the decomposition of organic matter and the oxidation of ammonia in soil, thereby boosting the content of available nitrogen and phosphorus and enhancing soil quality [42]. *Candidatus Eremiobacteraeota bacterium* also promotes soil organic phosphorus decomposition and improves soil phosphorus cycling capacity [43]. The present study found that the rhizosphere soil of TM had a significantly higher abundance of *Solirubrobacterales bacterium*, *Capillimicrobium parvum*, *Mycobacterium colombiense*, *Mycobacterium rhizamassiliense*, and *Conexibacter woesei* were all significantly higher in abundance than CK. It is clear that aerospace mutagenesis increased the number of *Solirubrobacterales bacterium* in soil, enhanced carbon metabolism, and improved soil microbial colonization. Moreover, the elevated presence of *Capillimicrobium parvum*, *Mycobacterium colombiense*, *Mycobacterium rhizamassiliense*, and *Conexibacter woesei* augmented the ability of microorganisms in the rhizosphere soil of tea trees to decompose organic matter, thereby enhancing soil nutrient cycling, increasing the content of soil available nutrients, promoting the growth of tea trees, and ultimately increasing tea yield.

### 2.7. Functional Prediction and Intensity Analysis of Characteristic Microorganisms

**Figure 7:**
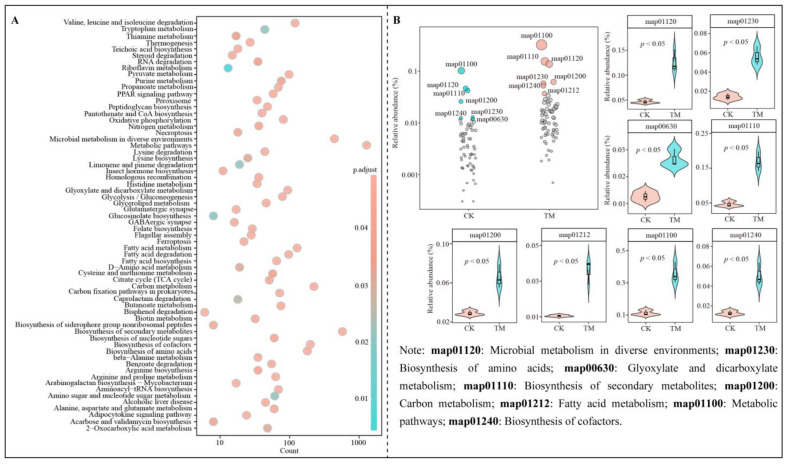
Functional prediction and intensity analysis of characteristic microorganisms. Note: CK is tea trees without aerospace mutagenesis; TM is tea trees with aerospace mutagenesis; (**A**) Functional enrichment of characteristic microorganisms; (**B**) Characteristic metabolic pathways screened by bubble feature map and their intensity analysis.

Based on the previous analysis, functional prediction and enrichment analysis were conducted for the six characteristic microorganisms. The results showed that these characteristic microorganisms corresponded to 4034 genes, which were annotated with various functions, and a total of 122 pathways were enriched, 64 of which were significantly enriched (Figure 7A). To further screen for characteristic pathways distinguishing TM from CK, bubble feature maps were employed. The results revealed that 8 characteristic pathways could significantly differentiate TM from CK (Figure 7B). These pathways included metabolic pathways (map01100), biosynthesis of secondary metabolites (map01110), glyoxylate and dicarboxylate metabolism (map00630), microbial metabolism in diverse environments (map01120), carbon metabolism (map01200), fatty acid metabolism (map01212), biosynthesis of amino acids (map01230), and biosynthesis of cofactors (map01240). Notably, the abundance of all 8 characteristic pathways was significantly higher in TM compared to CK.

Previous studies have suggested that high soil microbial diversity and rapid multiplication rates are beneficial for intensifying metabolic pathways and the biosynthesis of secondary metabolites in soil microorganisms [44]. It is evident that the microbial metabolic intensity and colonization rate were higher in the rhizosphere soil of TM compared to CK. However, the process of microbial reproduction requires the supply of raw materials as well as energy, and different types of enzymes are required to catalyze the biotransformations or reactions involved in the reproduction process [45]. The present study discovered that the metabolic intensity of microbial metabolism in diverse environments, glyoxylate and dicarboxylate metabolism, carbon metabolism, and biosynthesis of cofactors was significantly greater in TM compared to CK. Microbial metabolism in diverse environments is linked to the soil microorganisms’ ability to access resources and influences their metabolic capacity [46]. The enhancement of glyoxylate and dicarboxylate metabolism and carbon metabolism favors the release and accumulation of energy, which is advantageous for increasing the soil’s C-cycling capacity, reducing the accumulation of soil hazardous substances, and boosting microbial reproduction [47,48]. The cofactor products of the biosynthesis of cofactors pathway facilitate the activation of enzymes and reactants, accelerating enzyme-catalyzed reactions and the metabolic strength of various pathways [49]. It is evident that TM rhizosphere soil microorganisms possessed a greater ability to acquire resources and engage in carbon metabolism compared to CK. This provided more raw materials and energy for soil microorganisms to reproduce. Furthermore, the enhanced cofactor synthesis capacity of TM rhizosphere soil microorganisms facilitated more effective binding of enzymes to reactants and accelerated the reaction rate, which, in turn, promoted the multiplication of soil microorganisms and enhanced their functions. Additionally, this study also found that TM exhibited significantly higher capacity for fatty acid metabolism and biosynthesis of amino acids than CK. The products of the fatty acid metabolism pathway play a crucial role in regulating microbial community structure, favoring microbial colonization and enhancing microbial diversity [50]. The improvement in the biosynthesis of amino acids is beneficial for enhancing nitrogen mineralization, promoting an increase in soil available nitrogen, accelerating microbial reproduction, and fostering plant growth [51]. Therefore, it is evident that after aerospace mutagenesis, the reproductive capacity of the characteristic microorganisms in the rhizosphere soil of tea trees was enhanced, which subsequently increased the microbial diversity of the rhizosphere soil. This is conducive to promoting the conversion of soil nutrients, facilitating the uptake of nutrients by tea trees, and ultimately affecting the growth of tea trees.

### 2.8. Interaction Analysis of Characteristic Microorganisms and Their Functions with Different Indices

**Figure 8:**
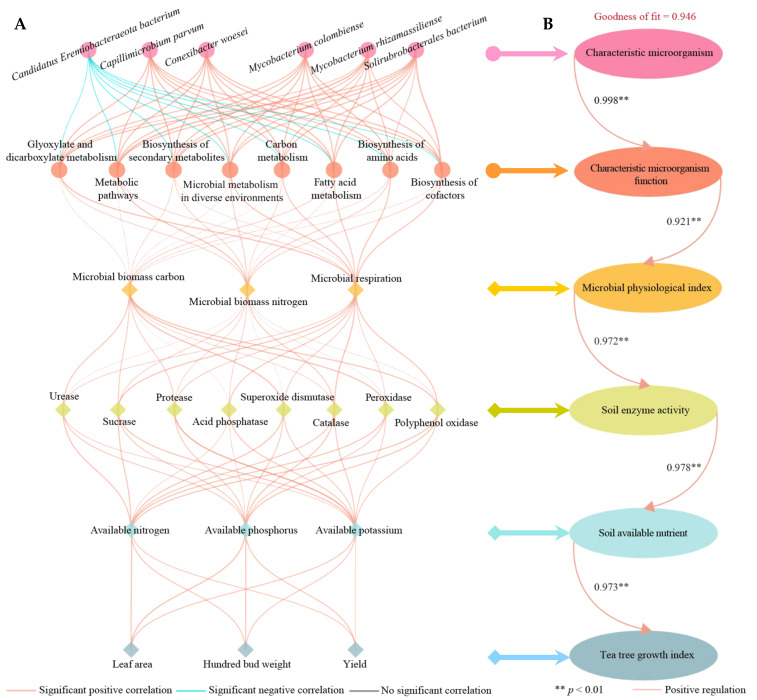
Correlation network and PLS-SEM equation analysis between different indices. Note: (**A**) Correlation network analysis; (**B**) PLS-SEM equation analysis.

Based on the previous analysis, this study conducted a further examination of the interactions between the characteristic microorganisms in tea tree rhizosphere soil and their functions with various indices. The correlation network analysis indicated that the characteristic microorganisms, specifically *Solirubrobacterales bacterium*, *Capillimicrobium parvum*, *Mycobacterium colombiense*, *Mycobacterium rhizamassiliense*, and *Conexibacter woesei*, exhibited significant positive correlations with soil microbial functions (Figure 8A). Conversely, *Candidatus Eremiobacteraeota bacterium* showed a significant negative correlation. The characteristic microbial functions were also significantly and positively correlated with soil microbial physiological indices, enzyme activities, available nutrient contents, and tea tree growth indices. Further construction of PLS-SEM equations between the characteristic microorganisms and different indices revealed that characteristic microorganisms positively regulated soil microbial function (0.998 **), and soil microbial function positively regulated soil microbial physiological indices (0.921 **), soil enzyme activity (0.972 **) and soil available nutrient content (0.978 **), which then positively regulated tea tree growth (0.973 **) and improved tea yield (Figure 8B). It is evident that aerospace mutagenesis increased the abundance of soil characteristic microorganisms in the rhizosphere of tea tree, enhanced the function of these microorganisms, and subsequently increased soil microbial biomass nitrogen, carbon, and respiratory strength, enhanced the activity of soil enzymes, and increased the production of soil available nitrogen, phosphorus, and potassium, which promoted tea tree growth and tea yield.

## 3. Materials and Methods

### 3.1. Experimental Site and Sample Sampling

The research was conducted at the Tea Tree Aerospace Breeding Experimental Base in Wuyishan, Fujian, China (117°59′47.7″ E, 27°44′8.4″ N). The subject of the study was the 11-year-old Dahongpao tea tree cultivar (*Camellia sinensis*). On 1 November 2011, tea tree seeds were sent into space with the “Shenzhou VIII” unmanned spacecraft to carry out space mutagenesis. After a total of 16 days, 13 h, and 34 min in space, the mutagenesis process concluded, and the capsule successfully returned to Earth on 17 November 2011. In April 2012, mutagenized and un-mutagenized tea tree seeds were germinated and planted in indoor pots. Due to the scarcity of mutagenized tea tree seedlings, they could not be used for a large number of research work to be carried out; therefore, the aerospace mutagenized tea tree seedlings were asexually propagated with the control, using cuttings in April 2016 to expand the planting area for further research and analysis. Tea tree seedlings obtained by asexual propagation were planted separately into open soil in different plots in the same area for uniform management. The size of each planting plot was 100 m^2^ (10 m × 10 m), and the spacing between different plots was 8 m, with an isolation fence in the middle. The soil used for planting tea trees was an acidic red loam with a pH value of 5.12. The basic physicochemical indexes of the soil were 1.02 g/kg for total nitrogen, 1.35 g/kg for total phosphorus, 1.68 g/kg for total potassium, and 7.45 mg/kg for available nitrogen, 11.83 mg/kg for available phosphorus, and 153.24 mg/kg for available potassium, respectively.

In October 2023, growth indices such as leaf area, hundred bud weight, and yield were measured for both tea trees that underwent aerospace mutagenesis (TM) and those that did not (CK). Additionally, rhizosphere soil samples were collected from CK and TM for analysis of soil available nutrient content, enzyme activity, microbial physiological indices, and macro-genome sequencing. The sampling procedure for the tea tree rhizosphere soil, as outlined in Wang et al. [52], involved randomly selecting five tea trees from CK and TM, respectively, removing surface residues from the soil at the planting site, gently excavating the tea trees, shaking off loosely adhering soil, and collecting the soil that remained tightly adhered to the root system. This soil was thoroughly mixed to obtain the rhizosphere soil sample. Three independent replicates of rhizosphere soil were collected for both CK and TM.

### 3.2. Determination of Growth Indices of Tea Trees

The growth indices of tea trees were evaluated following the methodology described by Ye et al. [53], focusing on leaf area, hundred bud weight, and yield. To determine leaf area, five secondary leaves were randomly selected from each tea tree, and their lengths and widths were measured. The leaf area was calculated using the formula (leaf length × leaf width × 0.7) and averaged to obtain one replicate; this process was repeated three times independently for each sample. Hundred bud weight was assessed by randomly collecting 100 buds and weighing them; this was performed in triplicate for each sample. Tea yield was measured by randomly selecting a 10 m^2^ area with planted tea trees, harvesting tea leaves according to the tea picking standards, and converting the yield to per hectare; this was also performed in triplicate for each sample.

### 3.3. Determination of Soil Available Nutrient Content

In this study, the soil’s available nutrient content was analyzed primarily for available nitrogen, phosphorus, and potassium using the methods outlined by Jia et al. [54]. Specifically, available nitrogen was determined by NaOH leaching followed by hydrochloric acid titration. Available phosphorus was measured using NaHCO_3_ leaching combined with molybdenum antimony resistance colorimetric method. Finally, available potassium was assessed by neutral ammonium acetate leaching and flame photometric method.

### 3.4. Determination of Soil Enzyme Activity

In this study, the activities of soil enzymes related to nutrient cycling and antioxidant defenses were evaluated using an Enzyme-Linked Immunosorbent Assay Kit sourced from Shanghai Preferred Biotechnology Co., Ltd. (Shanghai, China). The enzymes associated with nutrient cycling included urease, protease, acid phosphatase, and sucrase. For antioxidant enzymes, superoxide dismutase, peroxidase, catalase, and polyphenol oxidase were measured. The procedure involved taking 0.5 g of fresh soil, extracting it according to the kit’s instructions, and measuring the absorbance of the extract using a multifunctional enzyme labeling instrument (BioTek Synergy2 Gene 5, Winooski, VT, USA). The absorbance readings, measured at 630 nm, 680 nm, 660 nm, 540 nm, 560 nm, 470 nm, 240 nm, and 430 nm, respectively, for the aforementioned enzymes, were then converted to enzyme activity units expressed as U/g, and each sample was assayed in triplicate [55].

### 3.5. Determination of Physiological Indices of Soil Microorganisms

In this research, microbial biomass carbon, microbial biomass nitrogen, and microbial respiration were measured following the methodology outlined by Schnecker et al. [56], with each sample replicated three times. The chloroform fumigation extraction technique was employed to determine soil microbial biomass carbon and microbial biomass nitrogen. Specifically, 3 g of soil samples were fumigated with chloroform for 24 h, subsequently extracted using 1 M KCl, and analyzed using a TOC/TN analyzer (TOC-l CPH/CPN, Shimadzu, Kyoto, Japan). Microbial biomass carbon was calculated by dividing the difference between the fumigated and unfumigated soil organic carbon contents by 0.38. Similarly, microbial biomass nitrogen was calculated by dividing the difference in total nitrogen content between fumigated and unfumigated soils by 0.54. Microbial respiration intensity was assessed using the alkali uptake method, which measured the amount of CO_2_ released from the soil per unit of time, expressed in mg CO_2_/kg·h.

### 3.6. Macrogenome Sequencing and Bioinformatics Analysis of Soil Microorganisms

Soil genomic DNA was extracted utilizing the Soil Genomic Extraction Kit sourced from Tiangen, Beijing, China, adhering strictly to the kit’s instruction manual. Prior to library construction, the purity and integrity of the extracted DNA were verified through 1% agarose gel electrophoresis, while its concentration was accurately measured using a Qubit 3.0 fluorometer (Thermo Fisher, Waltham, MA, USA).

Library construction was carried out using the NEB Next Ultra DNA Library preparation Kit (NEB, Ipswich, TA, USA), following the manufacturer’s guidelines meticulously. An amount of 1 μg of DNA was processed, where it was fragmented randomly to approximately 350 bp in length through the use of ultrasonic crusher (E200, Covaris, Woburn, MA, USA). Subsequently, the DNA fragments underwent a series of steps, including end-repairing, A-tailing, addition of sequencing adapters, purification, and PCR amplification, all in accordance with the kit’s protocol, to complete the library preparation. Once the library was constructed, an initial quantification was conducted using a Qubit 3.0 fluorometer (Thermo Fisher, Waltham, MA, USA). The library was then diluted to a concentration of 2 ng/μL. To assess the insert size of the library, an Agilent 2100 (Agilent, Santa Clara, CA, USA) was employed. Following this, real-time fluorescence quantitative PCR was performed to precisely quantify the library’s concentration, ensuring an effective concentration of greater than 3 nM to guarantee library quality. The qualified libraries were subsequently submitted for sequencing and analysis on the Illumina HiSep High-Throughput Sequencing Platform.

Upon completion of sequencing, the raw data underwent quality control with Fastp software (v 0.20.1), where sequences with more than 40% low-quality bases (quality score < 15), those containing N bases exceeding 5 bp, and reads shorter than 15 bp were removed. Additionally, Bowtie2 software (v 2.3.4) was utilized to align the processed sequences against the host database, employing parameters set to “-end-to-end”, “-sensitive”, “-I 200”, “-X 400” to eliminate potential host-derived sequences and obtain clean data [57]. MEGAHIT software (v 1.2.9) was then applied for assembly analysis of the clean data, with parameters configured to “K-min 35”, “K-max 95”, “K-step 20”, “min-contig-len 500”. Bowtie 2 software (v 2.3.4) was used to compare the assembled contigs to obtain unutilized PE reads, and then mixed assembly was performed, with all parameters set to “-I 200”, “-X 400”, and then gene prediction [58]. MetaGeneMark (v 3.38) was employed to predict the open reading frame (ORF) of contigs larger than 500 bp after assembly [59]. For ORF prediction, predicted genes less than 100 nt in length were removed [60], and then the CD-HIT software (v 4.8.1) was used for de-redundancy to obtain a non-redundant initial gene catalog [61]. Subsequently, Bowtie 2 software (v2.3.4) was employed to align the clean data to the initial gene catalog, allowing for the determination of the number of reads mapped to each gene in each sample. By calculating the number of reads and gene lengths obtained from this alignment, information on the abundance of each gene within each sample was derived [62].

Species annotations were derived from acquired genes. Initially, the gene catalog was aligned with sequences of bacteria, fungi, archaea, and viruses sourced from NCBI’s NR database (using Blastp with an e-value threshold of 1 × 10^−5^) through the Diamond software (v 0.8.10) [63]. Subsequent analysis of the annotated species was conducted using the LCA algorithm within MEGAN software (v 2.1), leading to the final species annotation information for the sequence [64]. To annotate gene function, the acquired gene catalog was compared to the KEGG database using DIAMOND software (v 0.8.10) (Blastp, evalue ≤ 1 × 10^−5^). The gene function was assigned based on the highest-scoring comparison, with the relative abundance at each functional level being equivalent to the summed relative abundance of genes annotated within that same functional level [65].

### 3.7. Statistical Analysis

Excel 2020 served as the tool for preliminary statistical analysis and the creation of traditional stacked plots based on the raw data obtained. Statistical significance for differences in different indices was determined using paired Student’s *t*-tests, with thresholds set at *p* < 0.05 and *p* < 0.01 for significance. For the remainder of the graphical representations and data analysis, Rstudio software (v4.2.3) and different R packages were utilized [66]. The α-diversity (containing Shannon, Simpson, Chao1, ACE) and β-diversity were calculated using the vegan software package (version 2.6.8). Specifically, the gghalves package (version 0.1.4) was employed for generating box plots, while ggVennDiagram (version 1.5.2) was used to create Venn diagrams for gene and species analysis. Additional R packages, including minpack.lm (version 1.2.4) for neutral community models, igraph (version 2.0.2) for modular network interaction plots, ropls and mixOmics for Orthogonal Partial Least Squares Discrimination Analysis (OPLS-DA) model construction comparing CK and TM, and ggplot2 (version 3.4.0) for bubble feature maps to identify characteristic microorganisms, were also leveraged. For the Technique for Order Preference by Similarity to Ideal Solution (TOPSIS) analysis of microbial contributions, dplyr (version 1.1.4) was used. For post-microbial function prediction, KEGG enrichment was conducted using clusterProfiler (version 4.10.0) from the R package suite. Furthermore, vegan (version 2.6.4), linkET (version 0.0.7.1), and plspm (version 0.4.9) were employed for redundancy analysis, correlation interaction network creation, and partial least squares structural equation modeling (PLS-SEM) equation construction, respectively, across different indices.

## 4. Conclusions

**Figure 9:**
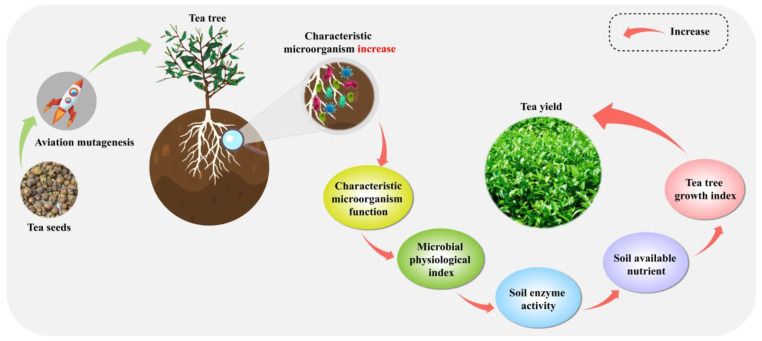
Mechanism analysis of the effect of rhizosphere soil microorganisms on tea tree growth.

In this study, we evaluate the impact of aerospace mutagenesis on tea tree growth, soil nutrient content, enzyme activity, microbial physiological indices, and microbial community structure and function. The results showed that compared with control, aerospace mutagenesis effectively increased leaf area and hundred bud weight of tea trees, resulting in increased tea yield. Additionally, aerospace mutagenesis significantly increased available nutrient content, soil enzyme activity, and microbial biomass carbon, nitrogen, and respiration intensity of tea trees. Microbial macrogenomic analysis further revealed that aerospace mutagenesis significantly increased the diversity and richness of microbial communities in tea tree rhizosphere soil, enhancing their diffusion capacity and leading to a more complex community structure. Notably, the key effect of aerospace mutagenesis was an increase in the abundance of five characteristic microorganisms in tea tree rhizosphere soil, namely *Solirubrobacterales bacterium*, *Capillimicrobium parvum*, *Mycobacterium colombiense*, *Mycobacterium rhizamassiliense*, and *Conexibacter woesei*. This, in turn, enhanced the activity of eight metabolic pathways in soil, including metabolic pathways, biosynthesis of secondary metabolites, glyoxylate and dicarboxylate metabolism, microbial metabolism in diverse environments, carbon metabolism, fatty acid metabolism, biosynthesis of amino acids, and biosynthesis of cofactors (Figure 9). Interaction network and PLS-SEM equation analysis showed that after aerospace mutagenesis, soil-characteristic microorganisms positively regulated microbial functions, positively regulated soil microbial carbon, nitrogen, respiration intensity, and soil enzyme activities, and then positively regulated soil available nutrient content and tea tree growth. In conclusion, aerospace mutagenesis increased the abundance of characteristic microorganisms in the rhizosphere soil of tea trees, significantly enhancing their functions. This, in turn, improved the soil’s nutrient conversion capacity, increased its available nutrient content, promoted tea tree growth, and increased tea yield. This study provides critical theoretical insights into the management of high-quality Dahongpao tea varieties following aerospace mutagenesis and microbial regulation of tea tree growth.

## Data Availability

Data will be made available on request. The original contributions of Metagenomic data presented in the study were publicly available. These data can be found here: NCBI, PRJNA1163912 (https://www.ncbi.nlm.nih.gov/bioproject/PRJNA1163912, accessed on 22 September 2024).

## Supplementary Materials

The following supporting information can be downloaded at: https://www.mdpi.com/article/10.3390/plants14070981/s1, Table S1 Basic information of soil macro-genome sequencing data. Table S2 Basic information of gene-assembled contigs obtained from each sample. Table S3 Basic information of Unigenes. Figure S1 Analysis of core and pan gene dilution curves for soil detected genes.
