# Supplementary material for: Aerospace Mutagenized Tea Tree Increases Rhizospheric Microorganisms, Enhances Nutrient Conversion Capacity and Promotes Growth
Source: Plants (Basel). 2025 Mar 21;14(7):981. doi: 10.3390/plants14070981 (PMC11990241; doi:10.3390/plants14070981)
Supplement: Supplementary file 1 [file plants-14-00981-s001.zip › plants-3523391-supplementary.pdf]

Supplementary Materials

Table S1 Basic information of soil macro-genome sequencing data

Sample	CleanData bases (G)	CleanData Q20	CleanData Q30	CleanData GC
CK-1	6.49	97.46	93.05	63.02
CK-2	6.87	97.52	93.19	63.3
C -3	6.53	97.47	93.09	63
TM-1	6.4	97.47	93.06	63.45
TM-2	5.86	97.47	93.1	63.5
TM-3	5.91	97.57	93.34	63.81

Note: CK: tea trees without aerospace mutagenesis; TM: tea trees with aerospace mutagenesis; Sample: Sample name; CleanData: Indicates valid data obtained by filtering; Bases: Number of bases in the data; Q20: Represents the percentage of the number of bases in the data with a sequencing error rate of less than 0.01(mass value greater than 20); Q30: Represents the percentage of the number of bases in the data with a sequencing error rate less than 0.001(mass value greater than 30); GC: GC content of bases in the data.

Table S2 Basic information of contigs after gene assembly obtained from each sample

Assembly	Sample					
	**CK-1**	**CK-2**	**CK-3**	**TM-1**	**TM-2**	**TM-3**
Contigs (≥ 0 bp)	245899	241255	201964	262730	234164	239133
Contigs (≥ 500 bp)	245899	241255	201964	262730	234164	239133
Contigs (≥ 1000 bp)	42431	33231	23146	42336	34782	38757
Contigs (≥ 5000 bp)	243	297	154	691	431	757
Contigs (≥ 10000 bp)	4	80	4	85	29	88
Contigs (≥ 25000 bp)	0	11	0	1	0	0
Contigs (≥ 50000 bp)	0	0	0	0	0	0
Length (≥ 0 bp)	197971342	186160292	147899213	212806740	183589264	194987153
Length (≥ 500 bp)	197971342	186160292	147899213	212806740	183589264	194987153
Length (≥ 1000 bp)	65311352	51680700	32932699	69124776	53876841	64940967
Length (≥ 5000 bp)	1498381	2707421	1007122	5075492	2955991	5446574
Length (≥ 10000 bp)	46376	1322478	47553	1106688	353311	1105368
Length (≥ 25000 bp)	0	328655	0	26296	0	0
Length (≥ 50000 bp)	0	0	0	0	0	0
Total contigs	245899	241255	201964	262730	234164	239133
Largest contig	12853	40927	14981	26296	18949	23223
Total length	197971342	186160292	147899213	212806740	183589264	194987153
GC (%)	62.09	62.95	62.78	63.48	63.72	64.32
N50	772	728	696	766	744	768
N75	598	585	574	598	591	596
L50	81173	82736	73664	85484	79467	76364
L75	154830	154783	132629	164943	149352	149274

Note: CK: tea trees without aerospace mutagenesis; TM: tea trees with aerospace mutagenesis; Sample: Sample name; Contigs: Indicates the number of contigs obtained by assembly; Length: Represents the length of the assembled contigs; Total contigs: The total number of contigs assembled; Largest contig: Indicates the maximum length of contigs; Total length: Indicates the total length of contigs assembled; N50(N75): Indicates that contigs are sorted by length, and then the sum is added from length to length, when the sum value reaches 50%(75%) of the total length of contigs; L50(L75): Indicates the number of contig when it reaches N50(N75).

Table S3 Basic information of Unigenes

List	Unigenes
ORFs number	462299
Integrity-start	108224 (23.41%)
Integrity-end	62734 (13.57%)
Integrity-none	146040 (31.59%)
Integrity-all	145301 (31.43%)
Total Length (Mbp)	636.34
Average Length (bp)	437.51
GC percent	64.16

Note：ORFs number: Indicates the number of genes in sample; Integrity-start: Represents the number and percentage of genes that contain only the start codon; Integrity-end: Represents the number and percentage of genes that contain only stop codons; Integrity-none: Represents the number and percentage of genes with neither start nor stop codons; Integrity-all: Represents the number and percentage of complete genes (both start and stop codons); Total Length: The total length of a gene catalogue; Average Length: Indicates the average length of genes in the gene catalogue; GC Percent: Represents the total GC content of the gene in the predicted gene catalogue.

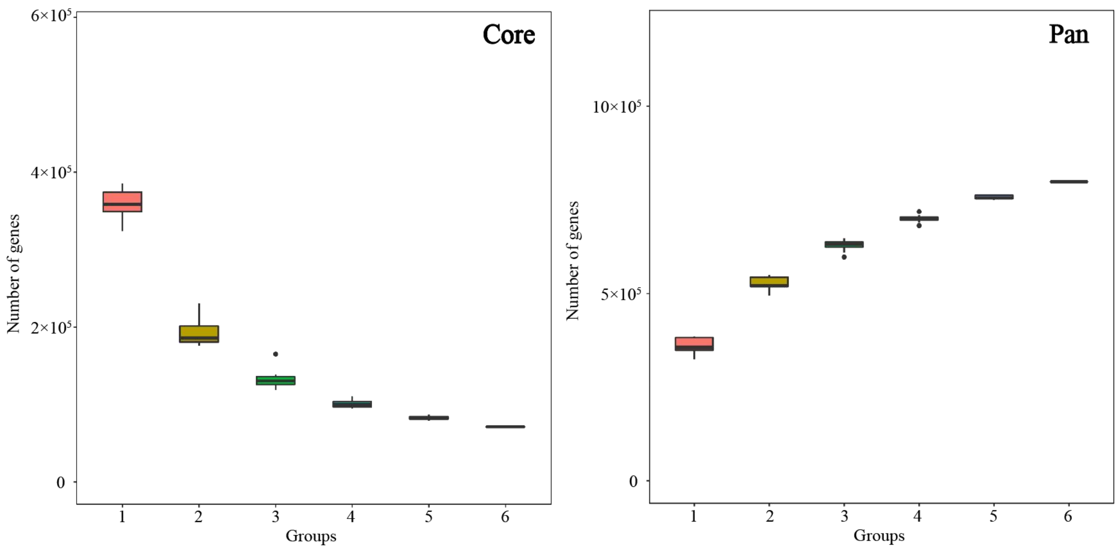

Figure. S1 Analysis of core and pan gene dilution curves for soil gene
